# Modified ABCDEF-Bundles for Critically Ill Pediatric Patients - What Could They Look Like?

**DOI:** 10.3389/fped.2022.886334

**Published:** 2022-05-02

**Authors:** Juliane Engel, Florian von Borell, Isabella Baumgartner, Matthias Kumpf, Michael Hofbeck, Jörg Michel, Felix Neunhoeffer

**Affiliations:** ^1^Department of Pediatric Cardiology, Pulmonology and Pediatric Intensive Care Medicine, University Children's Hospital Tübingen, Tübingen, Germany; ^2^Department of Pediatric Cardiology and Intensive Care Medicine, Hannover Medical School, Hannover, Germany

**Keywords:** pediatric critical care, post intensive care syndrome, PICS, ABCDEF-bundles, family centered care, PICUs (pediatric intensive care unit)

## Abstract

**Background and Significance:**

Advances in pediatric intensive care have led to markedly improved survival rates in critically ill children. Approximately 70% of those children survive with varying forms of complex chronic diseases or impairment/disabilities. Length of stay, length of mechanical ventilation and number of interventions per patient are increasing with rising complexity of underlying diseases, leading to increasing pain, agitation, withdrawal symptoms, delirium, immobility, and sleep disruption. The ICU-Liberation Collaborative of the Society of Critical Care Medicine has developed a number of preventative measures for prevention, early detection, or treatment of physical and psychiatric/psychological sequelae of oftentimes traumatic intensive care medicine. These so called ABCDEF-Bundles consist of elements for (A) assessment, prevention and management of pain, (B) spontaneous awakening and breathing trials (SAT/SBT), (C) choice of analgesia and sedation, (D) assessment, prevention and management of delirium, (E) early mobility and exercise and (F) family engagement and empowerment. For adult patients in critical care medicine, research shows significant effects of bundle-implementation on survival, mechanical ventilation, coma, delirium and post-ICU discharge disposition. Research regarding PICS in children and possible preventative or therapeutic intervention is insufficient as yet. This narrative review provides available information for modification and further research on the ABCDEF-Bundles for use in critically ill children.

**Material and Methods:**

A narrative review of existing literature was used.

**Results:**

One obvious distinction to adult patients is the wide range of different developmental stages of children and the even closer relationship between patient and family. Evidence for pediatric ABCDEF-Bundles is insufficient and input can only be collected from literature regarding different subsections and topics.

**Conclusion:**

In addition to efforts to improve analgesia, sedation and weaning protocols with the aim of prevention, early detection and effective treatment of withdrawal symptoms or delirium, efforts are focused on adjusting ABCDEF bundle for the entire pediatric age group and on strengthening families' decision-making power, understanding parents as a resource for their child and involving them early in the care of their children.

## Introduction

Within the last decade, long term complications after intensive care therapy have moved further into focus for both adult and, in later years, pediatric patients (1).

Measurement of outcome parameters in pediatric intensive care patients has been performed for decades, including the development of different scales and questionnaires like the Pediatric Overall and Cerebral Performance Categories (POPC, PCPC) and Functional Status Scale (FSS) (2, 3).

Using these tools, assessment of outcome after pediatric intensive care has shown a decrease in mortality from 5.8% in 1989/1990 to 4.6% published in 2000 down to 2.4% in 2014 (1, 3, 4).

In that same study, Pollack et al. (1) found the rate of significant new morbidities to be 4.8%, double that of mortality, and concluded “that pediatric critical care may have exchanged mortality for morbidity over the last several decades”.

In light of these developments, long-term survival and health related quality of life have moved further into focus.

Both the event leading up to the ICU-stay (congenital or acquired, traumatic or medical) and the repeated trauma caused by necessary interventions and therapies have long lasting effects on patients and their families. In adult patients, long-term consequences of intensive care treatment have been recognized as a relevant problem with an increasing focus on its prevention during treatment (5, 6). Lately, research and knowledge regarding pediatric patients and their families is increasing in this regard, reliable methods for prevention and treatment however are still lacking (7, 8).

The associated combination of debilitating symptoms following long-term or deep sedation, mechanical ventilation and forced immobilization has been identified and described by Needham et al. (5) as post intensive care syndrome (PICS). It includes significant physical (pulmonary, neuromuscular, and physical function), cognitive (“critical illness–related brain injury,” memory loss, lack of concentration, learning impairment) and mental/emotional (PTSD, fear, or anxiety disorder) problems and disorders which last long after discharge. Up to 64% of surviving adult ICU-patients without preexisting impairment suffer from one or more of these aspects (9). Extremely relevant for long-term outcome for instance is ICU-acquired weakness, characterized by symmetric myo-and polyneuropathy. It affects up to 67% of patients on mechanical ventilation at time of awakening and oftentimes persists after discharge (10).

Critically ill patients can present with problems from all categories. Additionally, relatives and caretakers often suffer from mental or emotional long term impairment such as anxiety or post-traumatic stress disorder (11). This phenomenon is described as PICS-F for “family” and affects relatives of up to 75% of patients (5, 12).

PICS in adult patients has been studied in depth and several projects have made it their goal to improve treatment and avoid its development altogether. A collaboration of intensive care professionals has developed the so called ABCDEF-Bundles, a number of measures meant to prevent PICS in both patients and their families (6).

They consist of several evidence-based treatment options meant to prevent or, if necessary, treat symptoms of PICS.

The ABCDEF-Bundles include (A) assessment, prevention and management of pain, (B) spontaneous awakening and breathing trials (SAT/SBT), (C) choice of analgesia and sedation, (D) assessment, prevention, and management of delirium, (E) early mobility and exercise and (F) family engagement and empowerment.

Since their first description, the bundles have emerged as a well-founded system for liberating patients from mechanical ventilation and improving long-term outcome.

Their implementation has been shown to be very effective in caring for critically ill adult patients, showing, among others, improvement in survival and disposition at time of discharge, reduction in time on mechanical ventilation, use of physical restraints and occurrence of delirium (13, 14).

As all elements are overlapping or interconnecting at some point, they have shown to be most effective when implemented together (13).

The successful implementation of these bundles and their increasing incorporation in routine adult critical care leads to the assumption that a similar paradigm shift in pediatric critical care is urgently needed.

A well-known problem in pediatrics is a delay in the introduction of new therapies for children (15).

Studies on ABCDEF-Bundles in children are still rare. Reliable recommendations for prevention and treatment of PICS in children have not been developed.

Studies show the effect of one to three bundles for use in children, confirming that scoring and treatment for delirium and early mobility can be successfully implemented with positive results (16).

A survey conducted in 15 European countries shows a high variation by region concerning implementation of individual bundles in pediatric intensive care units (17).

In 2020 Walz et al. (18) published a review regarding ABCDEF-Bundles for use in children. They conclude that ABCDEF-Bundles are suspected to be of similar use in children as in adults, even though clinical studies to their effect still need to be conducted. For all aspects they recommend establishing protocols and multidisciplinary teams for implementation of bundles in pediatric critical care. There are no further recommendations on changes that might need to be made in order to adapt the bundles for use in children (18).

The prevalent use of deep sedation and prolonged immobilization in treating critically ill children contributes to physical impairment like ICU-acquired weakness, mental problems following delirium and poor neurocognitive outcome.

In addition, Manning et al. (8) described one difference in PICS between adult and pediatric patients concerning the involvement of families. Besides known emotional and mental disorders such as PTSD or anxiety, which occur in families of adult patients as well, families of critically ill children suffer severe challenges to social interactions which affect both parents and siblings in their daily life.

Following the well-known adage “children are not small adults,” ABCDEF-Bundles like any new therapies and methods, need to be adjusted for use in children, taking into account flexibility for a broad range of developmental stages.

There is scientific evidence for some aspects of the Bundles for use in children, for others further research is needed. In this publication, an overview of existing literature and methods is given as well as suggestions for further development.

## Materials and Methods

Based on a narrative review of the existing literature on PICS and ABCDEF-Bundles in adult and pediatric patients, ABCDEF-Bundles are reevaluated and adapted for use in children by adjusting them as much as possible according to existing scientific evidence.

Taking into consideration current scientific evidence on analgesia and sedation, mechanical ventilation, management of delirium, mobilization and family involvement, pediatric ABCDEF-Bundles are being developed for implementation in the treatment of critically ill children.

Data sources: A systematic search of PubMed database was undertaken for full articles pertaining to ABCDEF Bundle and PICS, case series, observational and cohort studies and randomized controlled trials were included.

Study selection: No language or date barriers were set. Studies that met the following eligibility criteria were included: The study design aimed to describe the prevalence of PICS and the causes resulting from critical care treatment, as well as the description and effectiveness of ABCDEF Bundle on outcome.

Data extraction: Data were extracted by the primary researcher and accuracy checked by coauthors.

Data synthesis: A narrative synthesis was undertaken.

## Results and Discussion

For adult patients in critical care medicine, research shows significant effects of ABCDEF- bundle-implementation on survival, mechanical ventilation, coma, delirium, and post-ICU discharge disposition.

For children, a recent survey showed an implementation of all aspects in only 9% of 161 PICUs in the US, Canada, Brazil and Europe (19). Although there are calls for implementation of these measures in pediatric intensive care, as with most medical developments, the use of adult therapies in children without crucial changes beyond adaptation for size and body weight has not been effective (18).

Therefore, adjustments are necessary where proven methods in adults do not show the same results in pediatrics.

Before addressing each of the elements, a framework for implementation needs to be established. In analyzing adherence to ABCDE-Bundles (not including “F” for family involvement and empowerment) the complexity of combined ABCDE-Bundles has been identified as one major obstacle to adherence to bundles. On first impression, bundles are associated with an increased workload within an already stressful work environment. Studies and reports to this effect are difficult to compare, as variables are not clearly defined and success or adherence is rated differently across publications (20).

Other aspects identified include concerns over patient stability or safety, providers lack of knowledge regarding reasons and goals behind bundles, unclear or difficult to follow protocols and lack of coordination within inter-professional teams (21).

In order to improve compliance with guidelines and facilitate implementation of bundles in daily critical care routines, structured and repeated training of all professionals involved is a necessity. Continuous reinforcement can be assured by establishing champions within the team, taking on responsibility for adherence to protocols and acting as intermediaries in case of doubt or questions as to the procedures. Protocols need to clearly define methods for assessment, prevention and treatment of symptoms, assign responsibility for different aspects to all professions involved and therefore dividing the burden of perceived increase in workload on many shoulders (20).

Clearly structured documentation within already established patient records without need for additional systems help monitor adherence as well as results and enable reevaluation and adjustment of bundles.

An analysis of ABCDEF-Bundle use in critically ill adult patients showed a dose and response effect, with an increase in effect dependent on the amount of bundle aspects implemented. While all aspects are at some point connected and have synergistic effects when used in combination, we therefore stipulate, that use of just some aspects should always be preferred over not using any at all because of limited resources (13, 16).

In 2016, Yaghmai et al. (22) demonstrated a deterioration in adherence to nurse-controlled sedation protocols after initial successful implementation, showing the need for continuous efforts in training and monitoring.

Considering the widely acknowledged problem of a “theory-to-practice gap” in all fields of academic study, including medical research, nursing science and others, the process of implementing some or all bundles should be guided by current recommendations from implementation science in order to reach permanent use and effectiveness (23, 24).

### Assessment, Management, and Prevention of Pain and Choice of Sedation

Guidelines on analgesia and sedation differ according to regions and availability of substances and protocols should be adjusted accordingly.

Disoprivan, for instance, is recommended for use in children for up to 48 h within the United States but its use is not allowed for long-term sedation in Europe because of risk for propofol infusion syndrome in children under the age of 16 (25, 26).

We advocate for nurse-controlled protocols primarily using opioid infusion supplemented by alpha-2-agonists. Additionally, non-opioid drugs should be used for mild to moderate pain without the need for further sedative effect (25). Spinal anesthesia has been shown to effectively reduce opioid use in pediatric postoperative patients and has a significant benefit in providing hemodynamic stability in infants after surgery (27, 28).

In both adult and pediatric care, assessment of pain can best be accomplished by self-reporting using the numeric rating scale or visual analog scale. Unfortunately, in pediatric intensive care patients are oftentimes unable to participate due to either severity of illness or physiological developmental stages. There are several Scores available for use in such cases, i.e., the FLACC-Score or, in German speaking countries, the so called KUS-Skala (kindliche Unbehagens- und Schmerzskala) (29). They are validated for use in children <4 years and can also be used in older children with neurologic or developmental impairment (30, 31). All these scales are scored with points between 0 and 10 with any score ≥4 being seen as a reason for intervention. For postoperative assessment of sedated and even intubated children of all ages the Comfort-B-Scale is also available (32).

In general, the choice of scoring tool is not as important as the fact of scoring at all. It is recommended to evaluate pain regularly, we suggest every 8 h or more often in case of manifest pain and after intervention. Additionally, children under continuous analgesia and sedation can be scored using non-verbal scales in an attempt to differentiate between pain and undersedation for more appropriate intervention (33).

Prevention of pain should be achieved by using analgesia before any kind of potentially painful procedures, including endotracheal suction, blood draws, or other routine interventions.

Closely connected to bundle “A” is bundle “C,” Choice of sedation, which can also be achieved by implementing a protocol for analgesia and sedation.

In order to reduce stress and anxiety of patients as well as the safety risk to patients dependent on mechanical ventilation and catheters, undersedation needs to be avoided. On the other hand, oversedation carries the risk of prolonged mechanical ventilation, hemodynamic difficulties and an increase in withdrawal and delirium.

The goal should be sedation by continuous drug infusion which provides for patients in comfort, who are tolerating mechanical ventilation but are awake enough to perceive some of their environment and to communicate any discomfort which may be eliminated without the need for further sedation. In older children and adolescents, communication via drawing or writing should be made possible if tolerated by the patients.

The goal in both children and adults is the prevention of over- and undersedation with the long-term effect of reduction in withdrawal and delirium. For children, midazolam has been shown to increase delirium, decrease quality of sleep and prolong both length of mechanical ventilation and length of stay in the PICU. Most importantly, they have emerged as an independent risk factor for the development of pediatric delirium (34). While not all studies show a reduction in length of mechanical ventilation after implementation of sedation protocols, they do show a decrease in days with pain, withdrawal or delirium (35, 36). Use of sedation protocols has been shown to help in reducing use of benzodiazepines, support the interdisciplinary communication in order to set and manage goals of sedation and to lessen the presentation of iatrogenic withdrawal symptoms (37).

Several studies have shown alpha-2-agonists like dexmedetomidine and clonidine to have a sedative effect leading to a reduction in opioid- and benzodiazepine-requirement. At the same time, they prove to be less neurotoxic than other substances and lead to a lower occurrence in withdrawal and delirium (38, 39).

Protocols therefore should call for the sparing use of benzodiazepines in critically ill children, using opiates and alpha-2-agonist clonidine for firstline treatment (25, 33). Even with our knowledge of side effects and negative long-term effects, there are still patients who are sedated using benzodiazepines. In 2018 Shildt et al. could show that even with successful implementation of a benzodiazepine-sparing protocol, 30% of patients received midazolam infusion after sedation was found to be insufficient. The authors discuss whether some of those patients might have suffered from undetected delirium and question the influence of the practitioners' comfort with established routines using midazolam (40).

For any protocol based on titration of dosage to the effective level, there can be a reluctance in timely reduction. Therefore, regular scoring should involve active reevaluation of possible oversedation and protocols should call for attempted reduction in calm children.

The problem of iatrogenic withdrawal syndrome after long-term sedation is not included within the original ABCDEF-Bundles for adult patients. In 2019, Arroyo-Nonoa et al. (41) found only 8 works on IWS in adult critical care patients, with two published between 1998 and 2016 and 6 between 2017 and 2019. In contrast, this is one aspect where pediatric research is more advanced, having introduced and validated scoring tools for early detection (42, 43), after showing it to lead to relevant stress for both patients and parents (19). Additionally, IWS has been shown to be an independent risk factor for development of delirium, which in turn factors heavily within the long-term effects of critical care treatment (see bundle D below).

Nurse driven protocols for analgesia and sedation including tapering schedules contribute to the reduction in IWS (44). We therefore recommend using standardized IWS-scoring at least every 8 h, for instance using the Sophia observation of withdrawal score (Sophia observation and withdrawal score—pediatric delirium in conjunction with delirium screening) (43).

A possible strategy for the avoidance of oversedation might lie within increased family involvement in taking care of mechanically ventilated patients where nurse-to-patient ratios do not suffice for individualized care.

### Both Spontaneous Awakening and Spontaneous Breathing Trials

A significant deviation occurs in adjusting bundle “B” for use in pediatric patients. In the original ABCDEF-Bundles for adult patients, “B” stands for “both spontaneous awakening and spontaneous breathing trials,” therefore tying it in closely between bundles A and C. It describes a standardized protocol for pauses in sedation and mechanical ventilation for assessing the patient while alert for any extubation readiness (6). In pediatric patients, a careful risk-benefit-analysis has to be performed. While mechanical ventilation is a vital part of critical care medicine, prolonged use brings with it risks such as need for deeper sedation, followed by hemodynamic instability, immobilization and infection, in turn leading back to a prolonged mechanical ventilation and length of PICU-stay (45–47).

Regular spontaneous awakening and breathing trials with pause in all sedation have been successful in reducing time on mechanical ventilation, length of PICU-stay and cumulative dosis of sedatives (45), but have not been proven effective in terms of short-term health related quality of life (48). Instead, compared to use of standardized sedation protocols with continuous reduction in sedation (40, 49), Vet et al. (50) showed daily sedation interruption in addition to protocolized sedation to increase mortality in critically ill children when compared to those under protocolized sedation only. There were no added benefits for clinical outcome in the combined group.

On the other hand, continuous titration of sedation might lead to a hesitancy in reducing sedatives after reaching a comfortable dosage and prolonging sedation, mechanical ventilation and length of stay (22). Likewise, it has been found that protocols for weaning from mechanical ventilation should include clear instructions for when to start reducing parameters in order to avoid unnecessary delay (51).

An early study on weaning and extubation readiness has shown a high percentage of children to be ready for extubation on their first extubation readiness test, suggesting a lack of extubation readiness tests in early stages of treatment and the danger of unnecessarily prolonged ventilation (52). Additionally, upper airway obstruction ranged as a main factor for extubation failure, which cannot be detected by spontaneous breathing trials (52).

However, for patients with congenital heart disease, spontaneous breathing trials and daily extubation readiness tests proved effective in reducing extubation failure and length of PICU-stay (53, 54).

We therefore propose focusing any aspects concerning analgesia and sedation within bundle A and renaming bundle B as “Breathing and mechanical ventilation” for use in pediatric critical care. We advocate for a proactive and continuous weaning protocol with standardized daily reevaluation of mechanical ventilation and assessment for weaning, regular reduction of ventilator parameters in conjunction with protocolized reduction in sedation once feasible and daily extubation readiness tests for identification for extubation as early as possible in hopes of further reducing time on the ventilator and maybe even length of stay on the PICU (52). In support of this goal, early use of non-invasive ventilation should be considered.

### Assessment, Management and Prevention of Delirium

Delirium is a significant complication in critically ill children consisting of several symptoms of acute cerebral dysfunction. It has been found in up to 66% of patients in PICUs and is associated with prolonged time on mechanical ventilation, higher use of sedatives and physical restraints and leads to an increase in mortality as well as a reduction of health-related quality of life (55–57).

As with pain and sedation, regular assessment by using validated tools is the key for adequate management. Delirium remains underdiagnosed and misinterpreted in children and therefore undertreated, especially as children in hypoactive delirium are often seen as just especially calm and “easy” to comfort (58). All children admitted to a PICU should be subject to routine screening for withdrawal and early detection of symptoms and diagnosis of both hypo- and hyperactive as well as mixed forms of delirium in children

Although there are several possible scoring systems, such as the widely used tools of the Cornell Assessment of Pediatric Delirium (CAPD) or the Pediatric Confusion Assessment Method—Intensive Care Unit (pCAM-ICU) (59, 60), there is advantage in using the Sophia observation withdrawal—pediatric delirium assessment (SOS-PD). It has been validated for use in all pediatric age groups and, more importantly, differentiates between symptoms of withdrawal and delirium (61).

There are several modifiable risk factors for delirium in critically ill children, including mechanical ventilation, use of benzodiazepines as long-term sedatives, physical restraints, noise pollution and a lack of adequate nutrition which need to be considered in treatment (55, 57, 62, 63). On the other hand, we have no influence on independent factors such as age, sex, or severity and type of illness (34).

Delirium bundles have already been developed and described in detail.

Early use of non-pharmacological measures such as helping the children to reorient themselves after sedation, providing glasses and hearing aids and toys from home can prevent development of delirium or go a long way in treating symptoms that have already manifested (64). One most promising aspect in prevention of delirium presents standardized analgesia and sedation, which aims at a reduction in dosage (especially concerning benzodiazepines and anticholinergic substances) and a shortening in length of sedation (65).

The most important factors include treatment within a calm and comforting environment, including the presence of pictures or toys from home and the continuous care by a parent or other close caregiver. Orientation (or reorientation) in space and time should be encouraged by use of hearing aids and glasses, clocks and calendars and upright positioning in bed where tolerated (66, 67).

In severe cases, using low dose antipsychotic drugs as off-label medication (i.e., Quetiapine, Levomepromazine) might be feasible, but high quality studies to their affect are still lacking. Available studies show a high risk of side effects like extrapyramidal symptoms and changes in corrected QT-time (68–70). After close consideration in each case, benefits may outweigh the risks and should not be discounted completely. Nevertheless, these results emphasize the importance of non-pharmacological treatment of delirium and the necessity of a change of culture in pediatric intensive care toward prevention of delirium in critically ill children (67).

Child life specialists and other specialists should be present on the ward in order to treat patients, support and educate families and help train all other staff in dealing with delirium in critically ill children.

### Early Mobility and Exercise

“E” stands for early mobilization in critically ill patients. It has been shown to have a positive effect on body function, reducing limitations on activity and improving muscle strength and ability to walk (71).

While literature shows a solid scientific foundation for adult patients, implementation in PICUs is lacking (19). Interestingly, within this field of study there are more reviews available than clinical studies (72).

Additionally, in a recent review Nydahl et al. analyzed 33 reviews concerning early mobilization in critically ill patients. Out of these, only 3 were analyzing studies concerning pediatric patients (72).

Restrictions in time and space, lack of personnel and fear of adverse events such as dislodging of endotracheal tubes or central venous catheters all present (real and perceived) barriers to mobilization of critically ill children (17, 73, 74).

Throughout the available literature, a timeframe for early mobilization is not clearly defined (75). For children however, both Wieczorek and Choong have defined early mobilization as starting within 72 h of admission, starting with assessment for mobilization within 24 h (76, 77).

Depending on severity of illness, state of sedation and clearly indicated restrictions in movement or instabilities because of trauma or surgery, mobilization can be implemented as passive, assisted active or active mobilization (72, 76).

As with all other bundles, key is establishing a reliable protocol for daily review of the patients' goals, clear documentation of reached milestones for continuity of care and a multidisciplinary approach including rehabilitation specialists, nurses, doctors and parents, communicating different perspectives and defining common goals in daily rounds. Having a standardized protocol instead of individualized plans is associated with improved outcome and lessens the risk of implicit bias in planning the therapeutic approaches (76, 78).

Reinforcing the importance of the last of the bundles, F for “Family”, family presence has shown a marked influence on successful out-of-bed-mobilization for children (aOR 7.83). Unfortunately, the same study showed about one quarter of patients in pediatric intensive care to be completely immobilized (17).

Several international initiatives advocate for standardized early mobility in children, making a solid case for implementation in all PICUs, showing it to be safe and feasible, even in low resource regions (17, 77).

### Family Engagement and Empowerment

Family education and empowerment are listed last within the concept of pediatric ABCDEF-Bundles but represent a key element. While PICS is relevant in critically ill children, trauma, or sudden illness of a child has a significant impact on all members of the household and other close relatives. Post-traumatic stress disorder, anxiety and sleep disorders in parents can disrupt daily life for the whole family for a long time after discharge, including for patients who leave the hospital without permanent impairment (8, 79).

In adult critical care, family engagement includes participation in rounds, ethics and palliative care consultations as well as the offer of being present during traumatizing situations such as CPR. All aspects have been shown to be beneficial for patients and family as well as staff (6).

A common reason for psychological long-term difficulties of family members is the parents' feeling of helplessness and lack of information experienced when coping with their child's illness (80).

In 2020 the EU PARK-PICU study evaluated family centered care by questioning if 24-h-presence by family members at bedside was possible (17). However, Meert et al. (81) described a much broader approach to family centered care in pediatric intensive care based on the recommendation by the American Academy of Pediatrics, calling for “an innovative approach to the planning, delivery, and evaluation of health care that is grounded in a mutually beneficial partnership among patients, families and providers that recognizes the importance of the family in the patient's life”.

Among the core aspects are open visitation hours for parents and individually prepared bedside visits by siblings. Even before the current pandemic led to restrictions in visitation all over the world, 24-h-attendence was made possible in about 88% of PICUs considering hospitals in the US, Canada, Brazil and Europe. Unfortunately, in Europe less than half of PICUs reported permitted 24 h presence by family members (19).

Apart from open doors and the theoretical possibility of being present 24 h a day, parents most benefited from having a place to sleep at the hospital and involvement in daily patient care as per their parental role as primary caregiver.

Another aspect are family centered rounds, including parents in case presentations and discussions at bedside after first informing parents of the purpose behind these rounds. Problems perceived by staff such as lack of teaching, inhibition regarding the discussion of difficult topics or prolonged time for rounds receded after adequate education among staff as to the benefits of family centered rounds.

Third, the offer of being present for traumatic events like CPR and for invasive procedures is included in family centered care. As with most other described aspects, education of all professionals involved and open communication with parents is key for success. Once those needs are met, family members overwhelmingly prefer being present and one study could show that parents present during CPR benefited by exhibiting less signs of intrusive thoughts, prolonged symptoms of grief and post traumatic avoidance behavior (82, 83).

One valuable tool is available in pediatric ICU-diaries (84). Adapted from adult intensive care as well, ICU-diaries enable parents to record impressions, information and feelings for later review and age-appropriate sharing with the patient after discharge. It has been shown to be beneficial to both parents and relatives for dealing with their own trauma as well as for patients in filling in gaps in memory (85).

Additionally, ICU-Diaries provided a helpful tool in not only enabling parents and patients to review and understand lived experiences, but also provided support in explaining difficult information to siblings and other relatives (86).

Unfortunately, current restrictions (in space and due to the pandemic) do not allow for 24-h presence of parents or other close relatives at the bedside. However, efforts should go toward lifting of any set visiting hours and allow extended presence of parents with children who are expected to benefit in terms of reduction of sedatives and improvement of psychological wellbeing. It must be taken into account that, for example, the presence of the family is most positively associated with mobilization out of bed, and probably with many other measures as well (17).

For long-term consideration, we propose further modifying and expanding the pediatric ABCDEF-Bundles.

A common denominator within all ABCDEF-Bundles is the topic of communication. Not exclusively regarding children but with a special emphasis within pediatric intensive care, parents and other caregivers are speaking for our patients and need to be included in all aspects of their care and relevant decision making. This necessitates a high level of information and discourse. Parallel to adult patients, communication with the patients themselves is another aspect needing consideration. And within any multiprofessional team such as those in critical care, communication between team members is of utmost importance. In a letter to the editor, Patak et al. (87) wrote “Perhaps communication should be a vital sign,” noting the lack of standardized assessment and documentation of patient communication. For further development of pediatric ABCDEF-Bundles we propose following their suggestion and renaming bundle “C” for Communication (87), including necessary education and training protocols for staff (37), standardized systems for information and discussion with parents including informational packets in different languages and reliable access to translator services where needed, implementation and further development of age appropriate communication strategies and tools for intubated or otherwise impaired critically ill children and providing support and infrastructure for calls and video communication with siblings and friends who are unable to visit. Considering the relevant psychological impact any trauma or severe illness has on parents, siblings and other relatives, psychologists should be an integral part of any PICU-team. Professional support for both patients and relatives might help early diagnosis and treatment of associated illness such as depression and anxiety and reduce long-term effects (12). Additionally, crisis-intervention training should be considered for any health care professional within the critical care setting.

The original “C” for choice of sedation will be incorporated into bundle A, being renamed “Analgesia and Sedation.”

Concerning this new bundle “C” as well as the established aspect “F,” several studies have looked at possibilities of health informational technologies in pediatric intensive care. Based on evidence, that most parents prefer receiving all information concerning their child's health as soon as it is available, rather than summarized at greater intervals, the effect of interactive monitors showing electronic health records for use by parents was evaluated and suggests an improvement in awareness for parents and support in informed decision making (88). In 2016, Brown et al. (89) also found the offer of electronic information tools within the ICU to be welcomed by both patients and relatives for receiving updated medical information for review.

Possibilities within this field seem endless, offering further options for improving individualized support for families, providing general as well as specific information and giving parents and caregivers the opportunity to review given information on their own terms and without the time constraints (real or perceived) often imposed on short updates by medical professionals.

In another bid for expansion of ABCDEF-Bundles in pediatrics, Choong et al. (76) mention “G” for good nutrition and H for humanistic medicine.

Without question, physiological nutrition, and healthy sleep patterns are fundamental needs for children recuperating from severe illness and to prevent further deterioration (90). For a newly developed bundle G we therefore propose to include, again, standardized protocols for daily reevaluation of nutritional needs, determining severity of illness, weighing parenteral against early enteral nutrition, defining caloric needs and identifying patients in need of rehabilitational specialists for assessment and treatment of feeding and swallowing difficulties (91, 92). Next to “Good nutrition” we include “good sleep” and promote early support of a circadian rhythm, moving any possible intervention and diagnostics aside from emergencies into daylight hours and providing a calm and dark environment for uninterrupted sleep during nighttime for all children, irrelevant of their depth of sedation (25).

**Figure 1:**
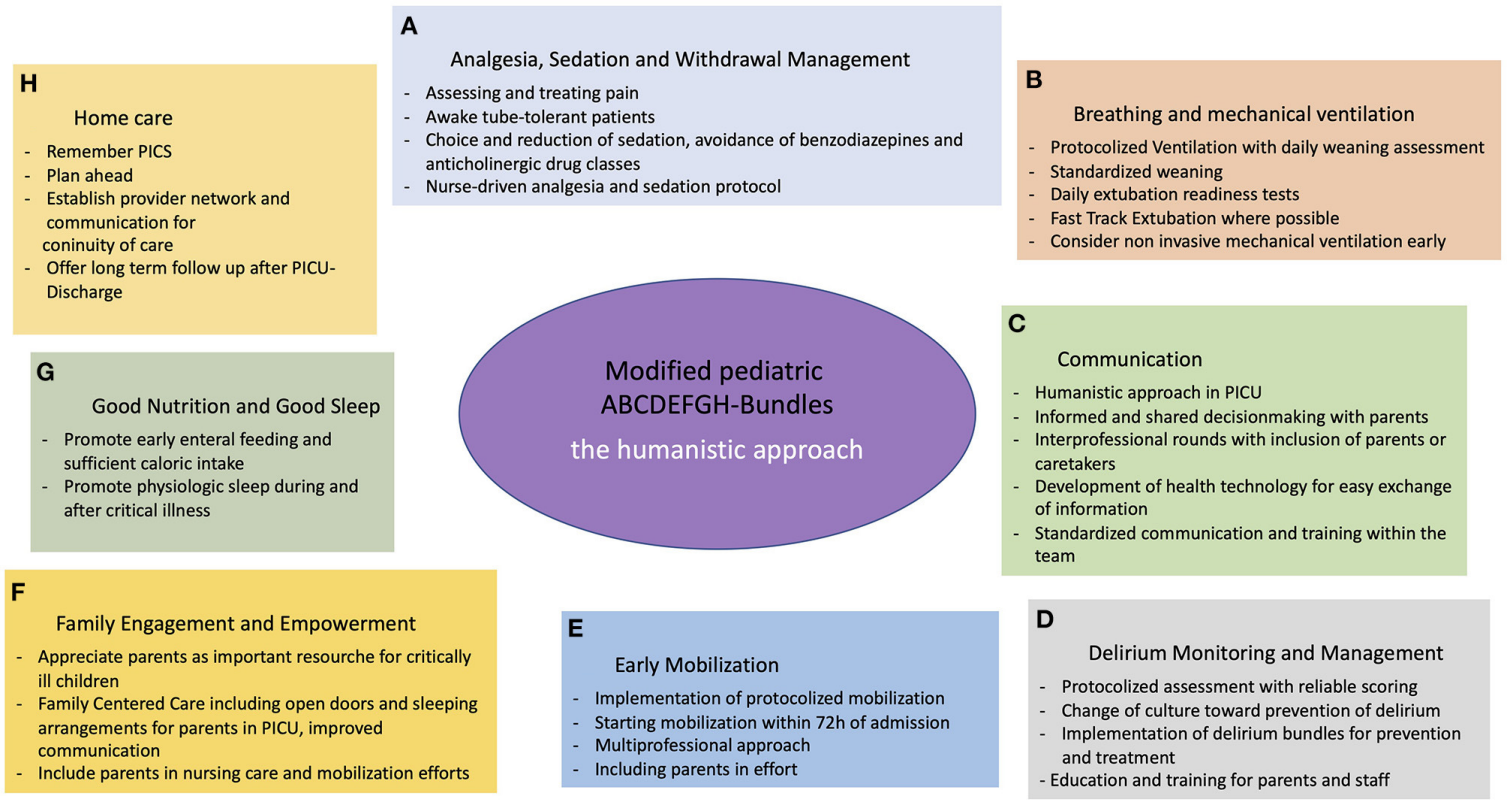
Modified pediatric ABCDEFGH-bundles—the humanistic approach. **(A)** Analgesia and Sedation; **(B)** Breathing and mechanical ventilation; **(C)** Communication; **(D)** Delirium Monitoring and Management; **(E)** Early Mobilization; **(F)** Familie Engagement and Empowerment; **(G)** Good Nutrition and Good Sleep; **(H)** Home care.

For a newly minted bundle “H”, however, we propose focusing on “home care,” using a humanistic approach throughout all bundles (Figure 1). For very few patients, their illness and treatment ends with discharge from PICU. Instead, more days on other wards within the hospital are often followed by ambulatory treatment and rehabilitation, including home care services, pediatricians and specialists, physiotherapy and many more. Other than during hospital stay, most of these different aspects often have to be coordinated by parents and caregivers themselves, which is made complicated by a scarcity of providers, especially in rural areas.

Long-term problems such as PICS or PTSD are often overlooked in these situations, leaving families without necessary support and treatment.

In order to ease this burden, we propose establishing follow-up services for parents and patients including screening for the development of PICS after discharge and coordinating services for rehabilitation specialists and other long-term health care providers. Comparable with ambitions in improving communication within the PICU-setting, this presents another aspect where health related technology should be developed, providing networking possibility and simplifying communication between providers for improved continuity of care after PICU discharge.

## Conclusion

While further studies are needed and in progress for the evaluation of long-term benefits of ABCDEF-Bundles in pediatric critical care, there is sufficient evidence for modifying existing ABCDEF-Bundles from adult care for use in children.

For all entities it is paramount to use written protocols which include scoring and daily assessment for early detection of either symptoms of withdrawal, delirium or pain as well as readiness for extubation, early mobility or other opportunities for progress without delay. Standardized interdisciplinary rounds including parents or other caregivers shorten delays in communication and provide parents with valuable information and insight in their children's illness and therefore empower them to actively participate in their improvement.

Key aspect is continuous training of all professionals involved in order to shorten time to diagnosis for both patients and families at risk for PICS and other long-term difficulties. Evidence-based findings should also be established more quickly and more comprehensively in daily routine care, keeping in mind, that successful implementation of only parts of the complete set of bundles already shows benefit for the long-term outcome and expansion of measures can occur gradually and in accordance with individual resources and recommendations from implementation sciences. The complex interaction between the elements and the fast-developing scientific evidence within the separate entities requires any health care provider in pediatric intensive care medicine to stay up to date and adapt therapies and guidelines accordingly.

## Author Contributions

JE, FN, and FB wrote the first draft of the manuscript. All authors contributed to the article and approved the submitted version.

## Funding

Open Access Publishing Fund of University of Tübingen.

## Conflict of Interest

The authors declare that the research was conducted in the absence of any commercial or financial relationships that could be construed as a potential conflict of interest.

## Publisher's Note

All claims expressed in this article are solely those of the authors and do not necessarily represent those of their affiliated organizations, or those of the publisher, the editors and the reviewers. Any product that may be evaluated in this article, or claim that may be made by its manufacturer, is not guaranteed or endorsed by the publisher.
